# *Faecalibacterium harmsenii* sp. nov.*,* an abundant but previously overlooked *Faecalibacterium* in the human gut

**DOI:** 10.1093/ismeco/ycag221

**Published:** 2026-07-31

**Authors:** Qiqi Pan, Eleni Tsompanidou, Wenbing Hu, Muhammad Tanweer Khan, Jan Maarten van Dijl

**Affiliations:** Department of Medical Microbiology and Infection prevention, University of Groningen, University Medical Center Groningen, Hanzeplein 1, Groningen, GZ 9713, the Netherlands; Winclove Probiotics, tt. Vasumweg 221, Amsterdam, SJ 1033, the Netherlands; Jiangxi Key Laboratory of Natural Products and Functional Food, College of Food Science and Engineering, Jiangxi Agricultural University, Nanchang 330045, China; Wallenberg Laboratory, Sahlgrenska University Hospital, Bruna Stråket 16, Gothenburg, SE-413 45, Sweden; Department of Medical Microbiology and Infection prevention, University of Groningen, University Medical Center Groningen, Hanzeplein 1, Groningen, GZ 9713, the Netherlands

**Keywords:** *Faecalibacterium harmsenii*, carbohydrate metabolism, defense mechanisms, mobile genetic elements, antibiotic resistance, ecological adaptation

## Abstract

*Faecalibacterium* is one of the most abundant anaerobes in the human colon. At the genus level, this bacterium shows a strong positive association with human health. Expanding collections of isolates and metagenome-assembled genomes have revealed its species diversity, yet species-level functions remain so far underexplored. Here, we describe a novel species, *Faecalibacterium harmsenii*. In addition, we reclassify another isolate as a member of the recently reported *Faecalibacterium langellae* species. Despite close genomic relatedness, these isolates exhibit distinct physiological and biochemical traits, including differences in carbohydrate utilization, stress tolerance, enzymatic activity, Gram-staining and fatty acid composition. Our present comparative genomics analyses further uncover extensive functional diversity and plasticity across type strains, with *F. harmsenii* being distinguished by an expanded carbohydrate gene repertoire and reduced defense systems, mobile genetic elements and antibiotic resistance genes. Extending to the species, we identify species-specific ecological niches across hosts and differential sensitivities to human diseases, highlighting certain species as reliable biomarkers of gut health. Together, these findings refine our understanding of *Faecalibacterium* diversity and provide a framework for its use in microbiome-based diagnostics and therapeutic development.

## Introduction

The gut microbiota is often described as the “second genome” of humans, comprising around 10^14^ bacteria that strongly influence host physiology [1]. These effects are mediated through various mechanisms, such as microbial metabolites [2, 3], secreted proteins [4–6], cell wall–associated molecules [7], interactions with the other gut microbiota [8, 9] and involvement in biotransformation processes [10, 11]. Most gut microbes are strict anaerobes, among which the genus *Faecalibacterium* is one of the most predominant bacteria [12].

The genus *Faecalibacterium* is well recognized as one of the major butyrate producers in the human gut, providing energy for colonocyte renewal and promoting gut barrier integrity [13, 14]. Beyond butyrate production, extracellular polymeric substances (EPS) from the *F. wellingii* strain HTF-F were shown to have an anti-inflammatory effect in a dextran sodium sulfate (DSS) induced murine colitis model [15]. Furthermore, culture supernatant of the *Faecalibacterium duncaniae* strain A2–165 exhibited an inflammation-suppressing effect in a 2,4-dinitrobenzenesulfonic acid (DNBS) induced murine colitis model through inhibition of the NF-κB pathway by its secreted microbial anti-inflammatory molecule (MAM) [6]. Extensive cohort studies have also positively linked a reduction of the *Faecalibacterium* genus to the status of human diseases, including diabetes [16], inflammatory bowel disease [17], obesity [18] and Parkinson’s disease [19]. Notably, *Faecalibacterium* has also been detected in other host backgrounds, such as chicken [20], piglets [21] and calves [22]. A study with piglets showed that outer membrane vesicles of *Faecalibacterium prausnitzii* DSM 107840 mitigated porcine epidemic diarrheal virus infection [23]. These findings highlight *Faecalibacterium* as a promising candidate for next-generation probiotics.

Despite extensive research, most functional studies have focused on the genus or strain level, or treated *Faecalibacterium* as a single species, namely *F. prausnitzii* [17, 24]. This oversimplification, common in earlier cohort studies, was largely due to the limited reference databases available at the time. With the expansion of cultured isolates and metagenome-assembled genomes, an increasing number of *Faecalibacterium* species have now been described [25–27]. However, their species-specific ecological distributions and functional contributions to host health have so far remained poorly understood. In our present study, we not only expanded the diversity of the genus by isolating and characterizing a novel *Faecalibacterium* species, but we also investigated the ecological distributions of existing species and evaluated their differential sensitivities to host disease conditions. Based on these analyses, we propose refined strategies for selecting species-specific biomarkers of gut health.

## Materials and methods

### Strain isolation and culture

*Faecalibacterium* sp. strains HTF-238 and HTF-05B were isolated from stool samples of healthy donors, as previously reported [28]. The type strain *F. duncaniae* A2–165 was kindly provided by Dr. Sylvia Duncan (Rowett Institute, Aberdeen, UK), and *F. prausnitzii* ATCC 27768 was obtained from DSMZ. All strains were revived from glycerol stocks and cultured in pre-reduced yeast–casitone–fatty acids-glucose (YCFAG) medium broth [28] at 37°C in a Bactron 600 anaerobic chamber (Sheldon Manufacturing, Inc., Cornelius, OR, USA) with an atmosphere of 10% H₂, 10% CO₂, and 80% N₂. Strain purity was verified prior to each experiment by MALDI-TOF MS (MALDI Biotyper® Sirius, Bruker, Fremont, CA, USA) using the previously described MALDIGut database [29].

### Bacterial morphological characterization

To characterize the bacterial cell morphology, 5 μl of overnight liquid cultures of strains HTF-238 and HTF-05B were evenly spread onto glass slides and dried on a heat block at 50°C. Gram staining was performed automatically using the PREVI® COLOR GRAM system (bioMérieux, Marcy-l'Étoile, France), and micrographs were obtained using a Leica ICC50 W microscope (Leica Microsystems, Wetzlar, Germany). Colony morphology on YCFAG plates was examined using a Leica M125 C stereomicroscope (Leica Microsystems, Wetzlar, Germany).

### Whole-genome analysis

Genomic DNA of strains HTF-238 and HTF-05B was extracted using the QIAamp DNA Microbiome Kit (Qiagen, Hilden, Germany) according to the manufacturer’s instructions. Whole-genome sequencing was performed on the Illumina MiSeq platform, generating 250 bp paired-end reads. Additional *Faecalibacterium* genomes used in this study were retrieved from the NCBI Genome database using the NCBI Datasets CLI (v16.x) on 2025-05-06 [30]. Raw reads were quality-checked and filtered using fastp (v0.20.1) [31], and assemblies were generated with SPAdes (v3.15.4) [32]. Assembly quality was assessed with CheckM (v1.2.2) [33]. *Faecalibacterium* genomes with >85% completeness and <5% contamination were retained for further analyses, with the exception of the designated type strains, which were included regardless of these thresholds. Gene prediction and annotation were performed with Prokka (v1.14.5) [34]. Genes related to short-chain fatty acid (SCFA) biosynthesis and the MAM protein were identified by Blastp (v2.13.0) using an e-value cutoff of 1e-3 [35]. A maximum-likelihood phylogenetic tree of the MAM protein was constructed in MEGA11 with 100 bootstrap replications [36]. Functional annotation of KEGG pathways was performed using BlastKOALA web server (https://www.kegg.jp/blastkoala/) [37]. Antibiotic resistance genes (ARGs) were identified using ResFinder (v4.7.2) (https://genepi.food.dtu.dk/resfinder) [38] with default cutoff values, and their genomic positions were determined with PlasFlow (v1.1.0) [39] using a filtering threshold of 0.7. Bacterial defense systems were annotated with the DefenseFinder web server (https://defensefinder.mdmlab.fr/) [40], and mobile genetic elements (MGEs) were identified using Blastp in DIAMOND (v2.1.8; e-value <1e-10) [41] against the mobileOG-db database (v2.0.1–90) [42]. Heatmaps of defense systems and MGEs were generated in R (v4.4.1) using the pheatmap package (v1.0.13). To quantitatively compare the abundance of functional genomic features across *Faecalibacterium* type strains, the numbers of MGEs, defense systems, and ARGs identified in each strain were summarized. For each functional category, the mean number across the type-strain panel was calculated. The difference between each strain and the corresponding group mean was then computed and visualized as a bar plot, with positive and negative values indicating numbers above and below the group mean, respectively. The plot was generated in R (v4.4.1) using ggplot2 (v4.0.0). The pan–core genome curve was constructed with Roary (v3.13.0), using a 95% identity threshold [43].

### Phylogenetic analysis

The 16S rRNA gene sequences of strains HTF-05B and HTF-238 were obtained by Sanger sequencing of PCR products using primers 27F and 1491R (Eurofins Genomics, Konstanz, Germany) [44]. The 16S rRNA sequences of the type strains of *Faecalibacterium butyricigenerans* and *Faecalibacterium longum* were retrieved from the China National GeneBank Database (CNGBdb) (https://db.cngb.org/), and all other 16S rRNA sequences were retrieved from the NCBI nucleotide database, with ID/assembly numbers provided in Table S1. Phylogenetic trees based on 16S rRNA were constructed using the neighbor-Joining method with 500 bootstrap replications in MEGA11 [36]. UBCG v2.0 was used to extract conserved genes and construct a core-gene tree with RaxML [45]. Average nucleotide identity (ANI) and digital DNA–DNA hybridization (dDDH) values between strains were calculated using BLAST+ in PyANI (v0.3.0-alpha) [46] and the Genome-to-Genome Distance Calculator (v3.0) web server (http://ggdc.dsmz.de/distcalc2.php) [47], respectively.

### Bacterial carbohydrate utilization measurement

Bacterial precultures grown in YCFAG were used to inoculate YCFA broth supplemented with one of the following carbohydrates: arabinose, cellobiose, sucrose, fucose, fructose, galactose, glucose, maltose, mannitol, mannose, lactose, raffinose, ribose, xylose, trehalose, rhamnose, or inositol. Each carbohydrate was supplied at a final concentration of 25 mM. Cultures were inoculated into 96-well plates at a 5% inoculum ratio. Growth was monitored for 40 h at 37°C by measuring optical density at 600 nm (OD_600_) at hourly intervals with an Epoch 2 microplate reader (Agilent Technologies, Santa Clara, CA, USA) housed in a Bactron 600 anaerobic incubator. The plate was shaken for 5 s at 237 cpm (4 mm amplitude) before each measurement, and all experiments were performed in triplicate.

### Bacterial growth condition measurement

Overnight precultures in YCFAG medium were used to inoculate YCFAG broth adjusted to different pH values (3.0–8.5, in 0.5 increments) or salinity levels (0%, 1%, and 2% NaCl) in 24-well plates at a 2% inoculum ratio with a final volume of 1.5 ml per well. The inoculated plates were incubated in an anaerobic chamber at 37°C, and OD_600_ was measured after 24 h using an Epoch 2 microplate reader (Agilent Technologies, Santa Clara, CA, USA). For growth assays at temperatures between 30°C and 45°C, 40 μl of glucose-grown precultures were used to inoculate 96-well plates containing 160 μl of YCFAG medium, and growth was monitored every 2 h for 24 h, using the same setup. The plate was shaken for 5 s before each measurement, and all experiments were performed in triplicate.

### Cellular fatty acid analysis

Bacterial pellets were obtained by centrifuging 1 l overnight YCFAG cultures at 4500 rpm for 10 min and resuspending them in 10 ml isopropanol. Fatty acid separation, detection, and quantification were carried out using the fatty acid methyl ester (FAME) method with GC-FID, and fatty acid components were identified by GC–MS on an Agilent 7000D system at the Leibniz-Institute DSMZ (Braunschweig, Germany) [48].

### Enzymatic activity assay

Bacterial enzymatic activity was assessed using the API ZYM kit (bioMérieux, Marcy-l’Étoile, France) according to the manufacturer’s instructions. Briefly, overnight cultures grown in YCFAG medium were harvested by centrifugation at 8000 rpm for 2 min, and the pellets were resuspended in 0.85% sterile NaCl solution. A 65 μl aliquot of the bacterial suspension was added to each cupule of the kit and incubated for 4.5 h in an anaerobic chamber at 37°C. After incubation, ZYM A and ZYM B reagents were added to stop the reactions. Color development was recorded after 5 min incubation at room temperature and scored according to the manufacturer’s guidelines for positive reactions.

### Metagenomic analysis

Metagenomic data from chicken, dog, cat, swine, cow, baboon, and human cohorts with inflammatory bowel disease (IBD) (including healthy controls), type 2 diabetes (T2D), and gestational diabetes mellitus (GDM) were retrieved from the NCBI SRA database. The BioProject numbers are shown in Table S2. Raw reads were quality-controlled using fastp (v0.20.1) [31], and host-derived reads were removed by mapping against host-specific reference genomes with Bowtie2 (v2.5.1) (Table S2) [49]. Taxonomic profiling was performed using Kraken2 (v2.1.2) with a custom database constructed from 11 representative *Faecalibacterium* genomes (Table S1) [50]. Species-level relative abundances were then estimated from the Kraken2 outputs with Bracken (v2.9), using a read length parameter of 150 bp [51].

## Results and discussion

### Phylogenetic analysis reveals the novel *Faecalibacterium harmsenii* species and reassigns strain HTF-238 to *Faecalibacterium langellae*

Two *Faecalibacterium* isolates from our laboratory, namely strain HTF-238 and HTF-05B, were previously classified as *F. prausnitzii*. However, both strains showed low similarity to the *F. prausnitzii* type strain ATCC 27768, with ANI values <95% and dDDH values <70% (Table S3). ANI values ≥95–96% and dDDH values ≥70% are commonly accepted thresholds for species boundaries. Therefore, we performed a comparison with all type strains officially assigned to the genus *Faecalibacterium.* This revealed that strain HTF-238 belongs to the recently described species *F. langellae* [27], with a dDDH value of 77.9% and an ANI value of 97.3% (Table S3). In contrast, strain HTF-05B was not closely related to any known species (Table S3) and it was, thus, identified here as a novel species that we named *F. harmsenii*.

**Figure 1:**
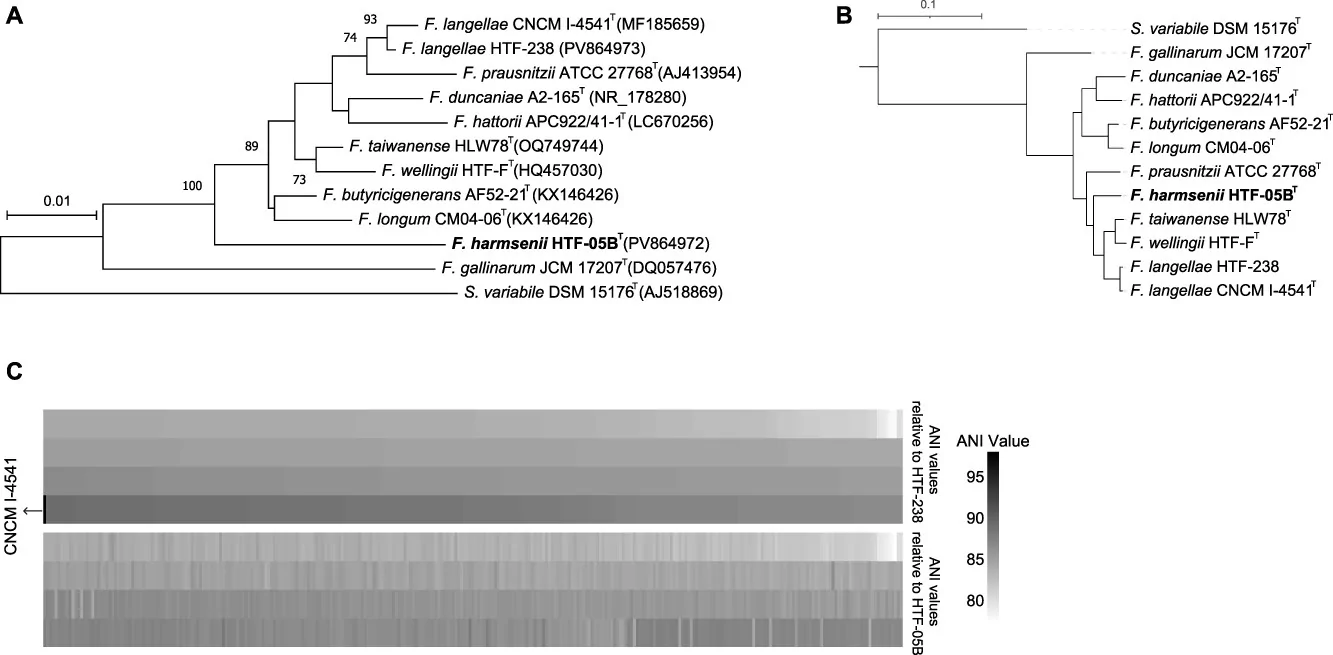
Phylogenetic and physiological features of *Faecalibacterium* isolates. A. Neighbor-joining phylogenetic tree based on 16S rRNA gene sequences. Bootstrap values (>70%) based on 500 replicates are shown at branch nodes. The scale bar indicates 0.01 substitutions per nucleotide position. B. Core gene phylogenomic tree of *Faecalibacterium* spp. constructed using the UBCG v2.0 dataset*. Subdoligranulum variabile* DSM 15176 was used as the outgroup in both phylogenies. C. ANI comparisons of HTF-238 and HTF-05B with other *Faecalibacterium* isolates. The upper block shows ANI values relative to HTF-238, and the lower block shows ANI values relative to HTF-05B.

Phylogenetic trees based on 16S rRNA and core genes consistently showed a distinct branch for strain HTF-05B and clustered strain HTF-238 with the *F. langellae* type strain CNCM I-4541, despite minor subclustering differences (Fig. 1A and B). Together, the observed phylogenetic and phenotypic distinctions support the recognition of a new species. Notably, the HTF-05B and HTF-238 strains, together with the *F. langellae* type strain CNCM I-4541, represent the only cultured members of the *F. harmsenii* and *F. langellae* species, respectively, based on ANI values across all publicly available *Faecalibacterium* genomes in the NCBI Genomes database (Fig. 1C). The difficulty of culturing strains from these two species may be explained by their limited growth on commonly used carbohydrate substrates, particularly evident in HTF-05B, which showed reduced peak biomass, prolonged lag phases (Fig. S1) and relatively strict carbon source requirements for growth (Table S4), including a narrow temperature tolerance.

### Distinct phenotypes of the novel *Faecalibacterium* isolates

**Figure 2:**
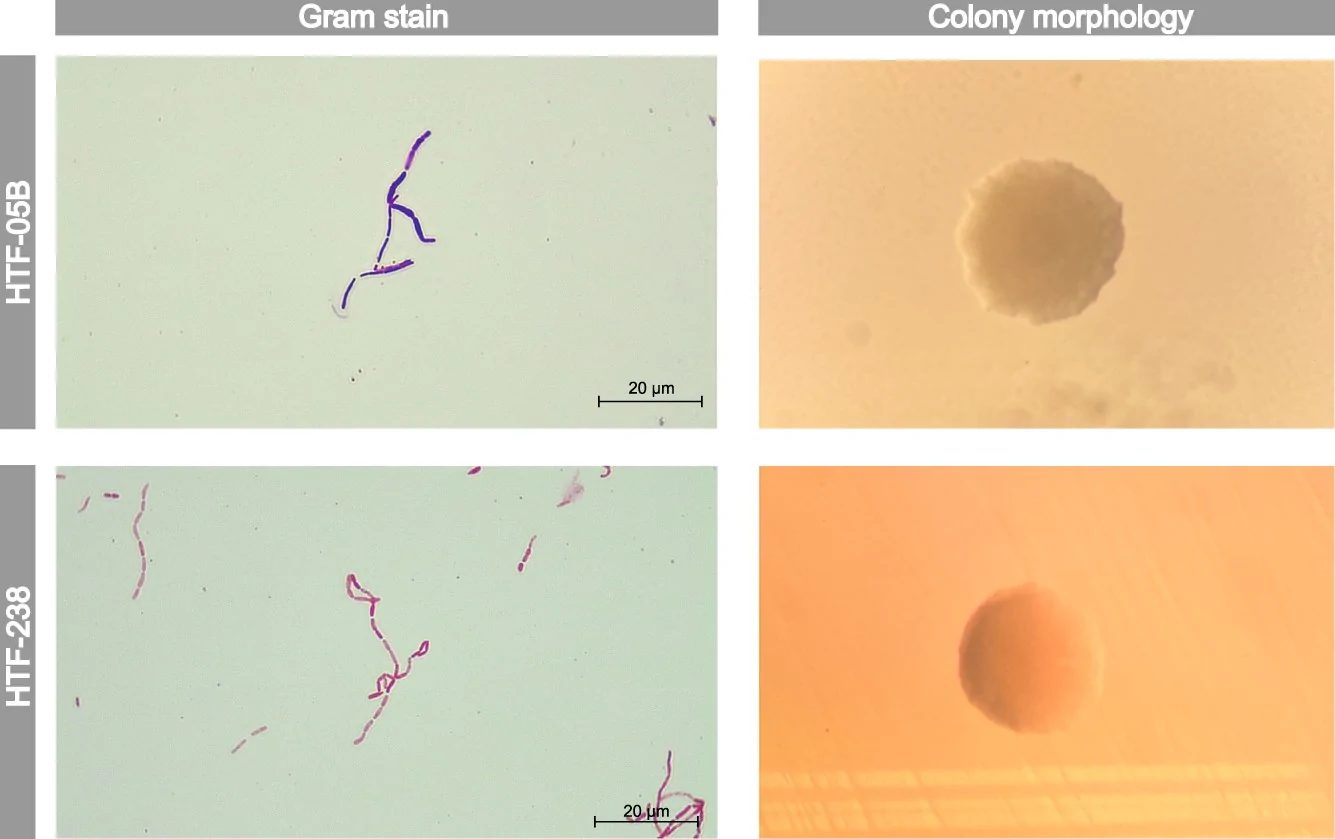
Colony and cell morphology of *Faecalibacterium* strains HTF-05B (top) and HTF-238 (bottom). The macroscopic colony morphology on YCFAG agar was observed at 25× magnification, and microscopic cell morphology was examined by Gram-staining at 1000× magnification.

Although *Faecalibacterium* species were initially described as Gram-variable [25], most recognized species consistently exhibit a Gram-negative staining [26, 27, 52]. In line with the previous report on the *F. langellae* type strain CNCM I-4541, *F. langellae* strain HTF-238 showed a Gram-negative phenotype (Fig. 2) [27]. In contrast, *F. harmsenii* strain HTF-05B reproducibly stained Gram-positive under the conditions tested in this study (Fig. 2), revealing a distinct Gram-staining phenotype. It should be noted here that the mechanism underlying the observed variability in Gram-staining of *Faecalibacterium* species is presently unclear.

*Faecalibacterium harmsenii* strain HTF-05B showed a cellular fatty acid profile that was broadly comparable to those of other investigated *Faecalibacterium* strains, particularly with respect to the presence of C16:0 and C16:0 DMA. However, the fatty acid profile of the HTF-05B strain differed in the relative abundance of C17:1ω6c and it lacked C18:1ω7c DMA among its major components (Table S5).

Additionally, compared with other phylogenetically related species, strain HTF-05B displayed positive reactions for β- and α-glucosidase, but tested negative for β-glucuronidase and esterase (C4) (Table S5). Notably, the two *F. langellae* strains differed in β- and α-glucosidase as well as β-glucuronidase activities (Table S5), underscoring the presence of strain-specific functional traits that should be considered in future studies.

Apart from these differences, the HTF-05B and HTF-238 strains shared a similar colony morphology on YCFAG agar after 48 h incubation, producing circular, convex colonies with undulate margins and opaque grayish coloration (Fig. 2).

### Comparative genomic analyses reveal functional diversity among *Faecalibacterium* type strains

**Figure 3:**
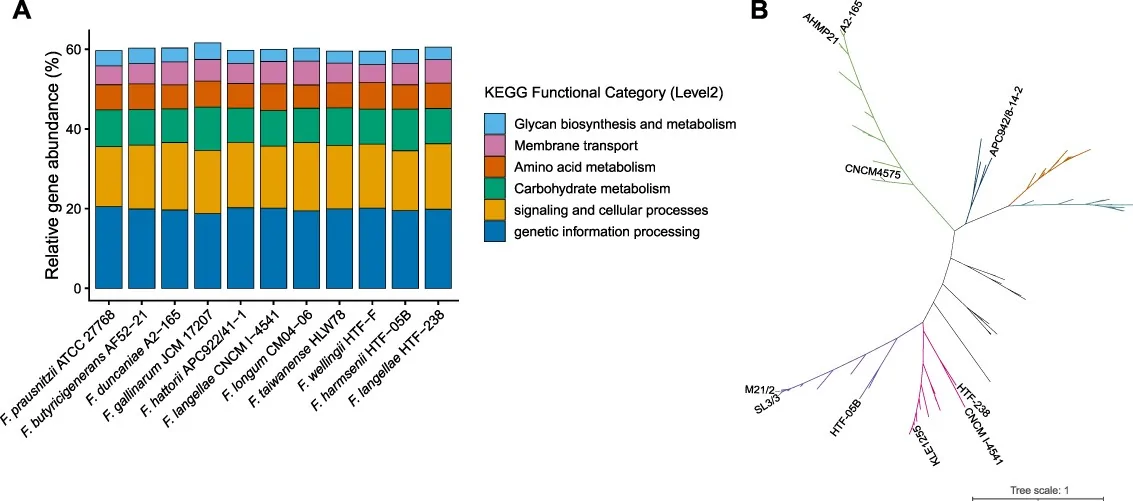
Functional gene distribution in *Faecalibacterium*. A. The most differentially abundant KEGG functional categories across 11 *Faecalibacterium* type strains. It should be noted that Fig. 3A shows only the most differentially represented KEGG functional categories rather than all KEGG-annotated genes. Therefore, the categories displayed do not sum up to 100%. B. Maximum-likelihood phylogenetic tree of MAM protein sequences. The scale bar represents one substitution per nucleotide position. Different colors indicate distinct clades. Anti-inflammatory properties of the labeled strains were tested in ref. [53].

The variation in genome size and GC content among *Faecalibacterium* strains suggests potential diversity in the distribution of functional genes (Table S1). To explore this further, we conducted a comparative genomic analysis and identified the top 20 most abundant KEGG functional categories in the *Faecalibacterium* type strains (Table S6). Across all strains, genes related to genetic information processing were most abundant, followed by genes involved in signaling and cellular processes, and carbohydrate metabolism. Notably, *Faecalibacterium gallinarum* JCM 17207 and *F. harmsenii* HTF-05B contained substantially more genes associated with carbohydrate metabolism and glycan biosynthesis than other type strains (Fig. 3A). These traits may broaden their ecological niches, especially given that JCM 17207 was originally isolated from chicken [26]. For HTF-05B, its broad carbohydrate utilization capacity observed in vitro (Table S4) further corroborates these genomic features. In contrast, the genome of the *F. langellae* strain HTF-238 encodes a relatively high number of genes linked to signaling and cellular processes, whereas the genome of the type strain CNCM I-4541 of the same species contains relatively fewer genes in this category (Fig. 3A), underscoring that *Faecalibacterium*’s genomic features can be highly strain-specific.

We next examined specific gene functions of interest. All type strains of *Faecalibacterium*, including the strain HTF-05B from the new *F. harmsenii* species, harbored the complete cluster for SCFA biosynthesis (data not shown), consistent with the genus-wide role in butyrate production [54]. However, strain-level variation in the SCFA production capacity has been observed experimentally [25–27], likely reflecting differences in transcriptional regulation. We also assessed the distribution of the MAM protein, a secreted anti-inflammatory factor in *Faecalibacterium* [6]. The gene encoding this protein was found to be conserved across all tested strains (Fig. 3B). Nevertheless, it has been reported that CNCM I-4541, despite carrying the MAM gene, failed to confer anti-inflammatory effects in animal models. This suggests that sequence variations may compromise the anti-inflammatory function [6, 53, 55]. Indeed, phylogenetic analysis showed that the MAM protein of CNCM I-4541 represents a distinct evolutionary branch compared to other strains, further supporting its divergence (Fig. 3B).

As *Faecalibacterium* is considered a promising next-generation probiotic, the presence of ARGs is a critical safety concern. Comparative analysis revealed variation in ARG content across type strains: *Faecalibacterium taiwanense* HLW78 carried the highest number of ARGs, followed by *F. longum* CM04–06 and *Frullania hattorii* APC922, whereas *F. harmsenii* HTF-05B, *F. prausnitzii* ATCC 27768, *F. gallinarum* JCM 17207, and *F. langellae* HTF-238 presented no ARGs (Table S7, Fig. S2). Importantly, all ARGs detected in the tested strains were chromosomally encoded (Table S7), limiting their potential for horizontal transfer to pathogenic bacteria.

### Genomic plasticity reveals prospects for genetic modification in *Faecalibacterium*

**Figure 4:**
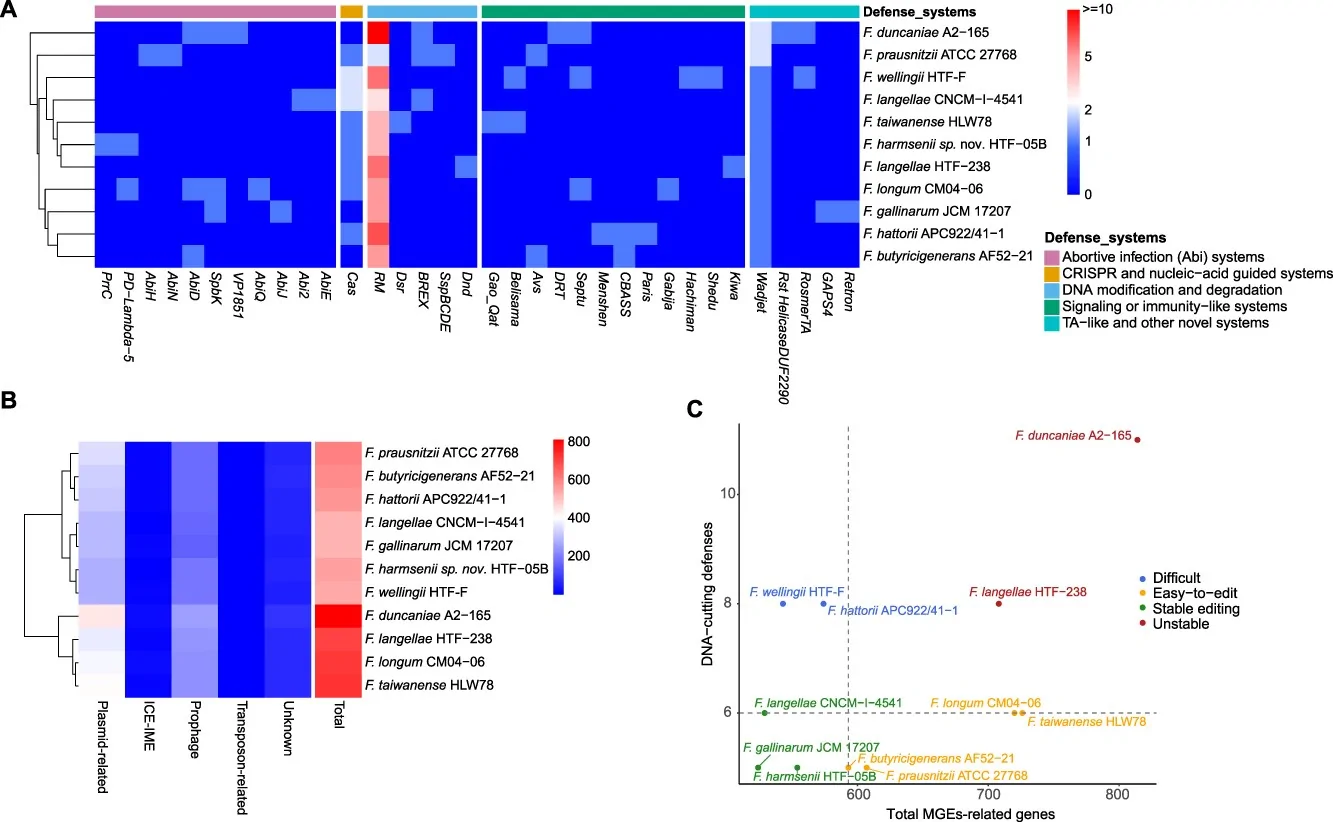
Genomic flexibility and plasticity of *Faecalibacterium*. A. Number of genes associated with bacterial defense systems. B. Number of genes related to MGEs. C. Predicted genome editing suitability of *Faecalibacterium* type strains based on the abundance of MGE-related genes and DNA-cutting defense systems. Each point represents one strain. Strains were classified into four predicted genome-editing categories according to the number of MGE-related genes and DNA-cutting defense systems encoded in their genomes, with dashed lines indicating the classification thresholds.

Despite its well-recognized contributions to host health, the lack of reliable genetic modification tools has hindered efforts to establish causal links between *Faecalibacterium* functions and host physiology. To assess the genetic tractability of the genus, we analyzed the distribution of bacterial defense systems against internalization of extracellular DNA and MGEs across all type strains. As shown in Fig. 4A, 4B and Fig. S2, *F. duncaniae* A2–165 encoded the highest number of both defense systems and MGEs. Among all categories, genes belonging to restriction–modification (RM) defense systems were the most abundant (Fig. 4A), which likely constrains the genetic plasticity of *Faecalibacterium*. Conversely, the high abundance of MGE-related genes could provide flexibility for the development of genetic tools (Fig. 4B), although excessive numbers of MGEs may also increase genomic instability.

Based on the combined abundance of MGEs and DNA-splicing defense systems, we classified strains into categories of genetic modification potential (Fig. 4C). In principle, strains with fewer defense systems and MGEs are expected to be more amenable to stable editing, whereas strains with extensive defense systems but few MGEs would be the most challenging ones to manipulate. According to this framework, *F. harmsenii* HTF-05B, *F. gallinarum* JCM 17207, and *F. langellae* CNCM I-4541 emerge as the most promising candidates for genetic modification among the type strains (Fig. 4C). Consistently, the quantitative comparison showed that these strains generally carry below average numbers of MGEs and defense systems compared with the type strain panel (Fig. S2).

Beyond basic research, the abundance of MGEs is also relevant for probiotic safety, as a high MGE content may increase mutation rates and can alter strain function in the host [56]. This consideration is particularly important for the development of next-generation probiotics capable of colonizing the human colon. Therefore, strains with relatively few MGEs, such as *F. harmsenii* HTF-05B and *F. gallinarum* JCM 17207, may represent safer candidates for probiotic development (Fig. 4C). However, it should be noted that the whole-genome sequence data used in this study were generated by short-read Illumina sequencing, which limits the resolution of MGEs, defense systems, and ARGs. Future long-read or hybrid sequencing analyses would help to better resolve these elements and their genomic contexts.

### Host-associated abundance patterns reveal distinct ecological niches of *Faecalibacterium* species

*Faecalibacterium* is one of the most abundant anaerobes in the healthy human gut microbiota, typically comprising ~5% of the total bacteria [12]. Nevertheless, its distribution in non-human hosts has rarely been investigated systematically. Here, we assessed species-level abundances of *Faecalibacterium* across different host guts by reanalyzing previously published metagenomic datasets (Table S2), using our custom *Faecalibacterium* database.

Across all hosts examined, *Faecalibacterium* was most abundant in healthy humans, followed by cats and the non-human primate baboon (Fig. S3A). Comparable abundances were observed in swine and chicken, whereas dogs consistently harbored low levels (<1%) (Fig. S3A). Notably, although *Faecalibacterium* was detected in calves, it was absent from adult cows.

**Figure 5:**
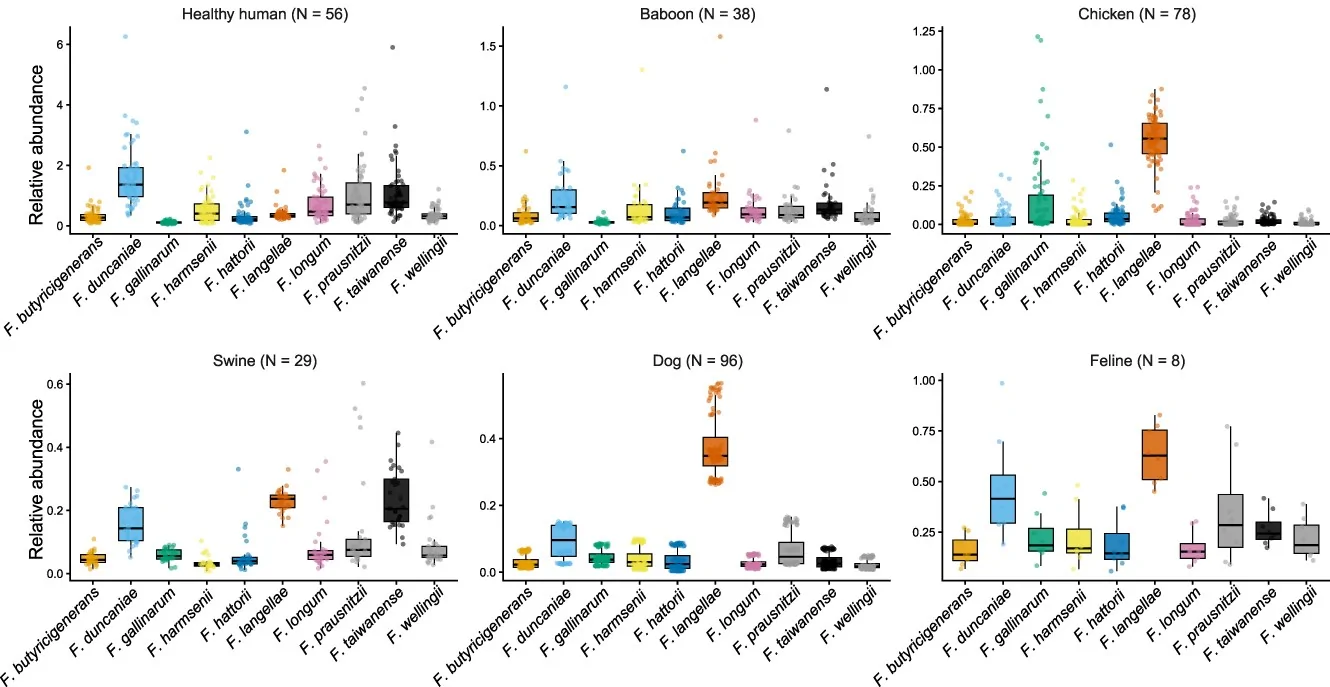
Ecological distribution of *Faecalibacterium* species across different hosts. Previously published metagenomic datasets were used to determine species-level relative *Faecalibacterium* abundance patterns within each host group. The respective BioProject numbers are listed in Table S2. Sample numbers for each host are indicated next to the corresponding host name in the figure. Of note, the use of data from different studies limits detailed comparisons between host groups.

At the species level, *F. duncaniae*, *F. prausnitzii*, and *F. taiwanense* were the dominant *Faecalibacterium* species in healthy humans, and a similar abundance pattern was observed in swine (Fig. 5). By contrast, *F. langellae* predominated in other hosts, including chicken, cats, and dogs (Fig. 5), highlighting the need to broaden the host range sampling to improve the isolation and characterization of host-associated *Faecalibacterium* diversity. Additionally, *F. harmsenii* remained at low abundance across all tested hosts, consistent with its strict carbon source and environmental condition requirements described above (Fig. S1 and Table S4). These host-dependent differences in the distribution of *Faecalibacterium* species are likely influenced by different dietary patterns and gut conditions. Therefore, strains isolated from non-human hosts should not be assumed to colonize the human gut efficiently without further functional validation, particularly if they are considered for future human-associated applications.

Although currently described *Faecalibacterium* species account for nearly all diversity within the genus across tested hosts (Fig. S3A), at least four additional undescribed species, each represented by multiple strains, were detected based on ANI differences (Fig. S3B). Furthermore, *Faecalibacterium* displays an open pan-genome, indicative of high genetic diversity and ongoing gene exchange within the genus, thereby increasing the likelihood of novel emerging species (Fig. S3C). However, here it should be noted that the applied k-mer-based tools, such as Kraken2/Bracken, may have some limitations in distinguishing highly similar or closely related genomes. Another potential limitation in our present analysis of host-associated *Faecalibacterium* abundance patterns is that the applied datasets were generated independently by different research groups and retrieved from public repositories. Thus, sample sizes, sampling procedures, sample processing, and storage conditions could not be fully matched across host species. Future cross-validation with complementary approaches would further strengthen species-level abundance estimates. In addition, larger cohort samples should be included in analyses on the ecological distribution of *Faecalibacterium* species to minimize potential bias.

### *Faecalibacterium* species display distinct disease-associated sensitivities

**Figure 6:**
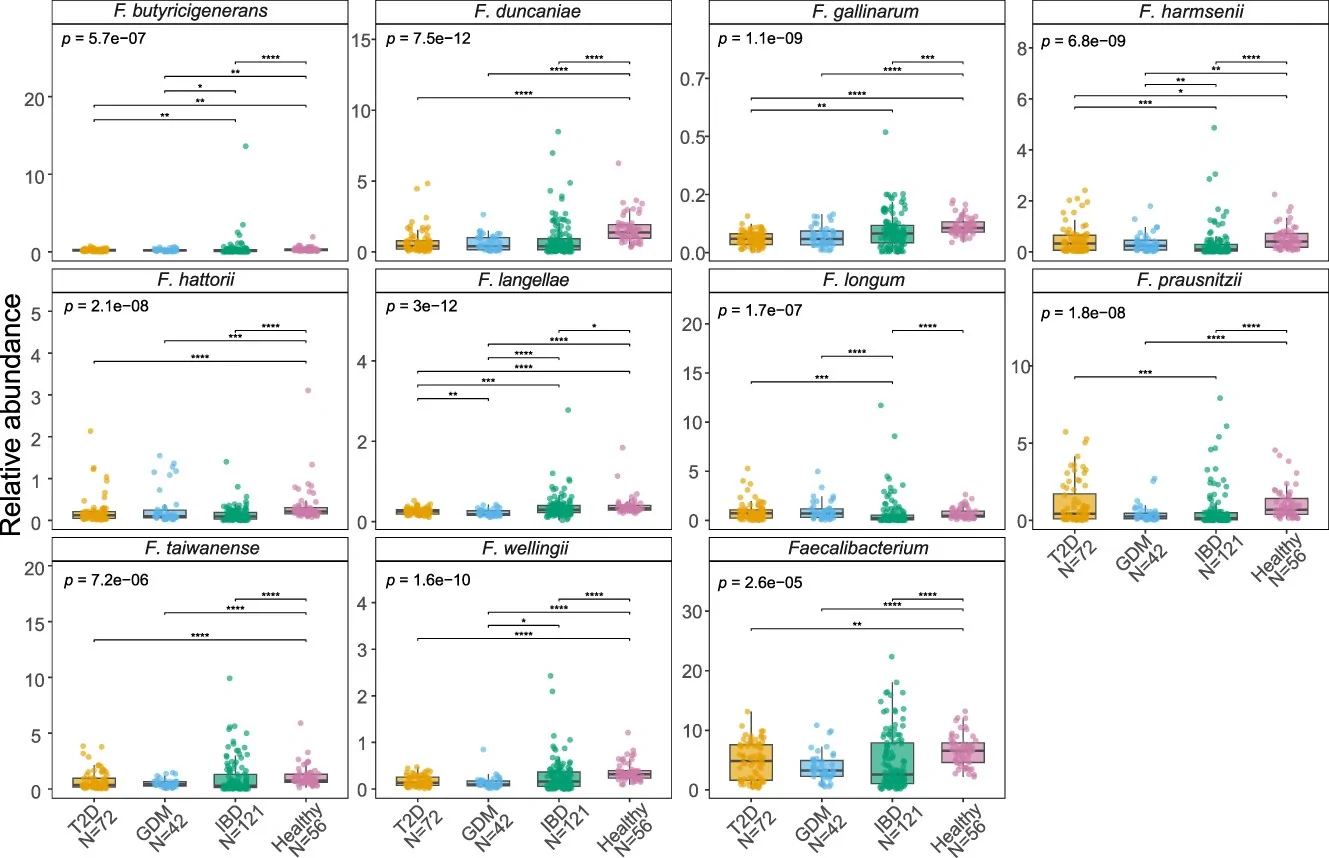
Relative abundance of *Faecalibacterium* species under different host physiological conditions. The metagenomic samples were obtained from previously published datasets, using the BioProject numbers listed in Table S2. Global differences were tested using the Kruskal–Wallis test. The *P*-value bars indicate pairwise post hoc comparisons between groups, annotated with Benjamini–Hochberg–adjusted *P* values from Dunn’s tests. Non-significant comparisons (*P* ≥ .05) are not shown. Please note that only the IBD cohort included healthy controls, while the T2D dataset mainly compared untreated T2D patients with treated T2D patients, and the GDM dataset involved pregnant women without GDM as controls. Abbreviations on the x-axis are defined as follows: T2D, type 2 diabetes; GDM, gestational diabetes mellitus; IBD, inflammatory bowel disease; healthy, healthy donors. The cohort data details are presented in Table S2.

The abundance of *Faecalibacterium* has often been used as an index of gut health in previous cohort studies [24, 57]. However, prior to its recent sub-classification, *Faecalibacterium* was solely identified as *F. prausnitzii*, representing the entire genus [17, 24]. Given the physiological and biochemical differences among species (Table S4), their responses to host physiological conditions may also be species-specific. In this study, we therefore examined species-level associations with diseases known to show reduced *Faecalibacterium* abundance, including T2D [16], GDM [58], and IBD [17]. As the IBD dataset was the only dataset that included an available healthy control group, this group was used as a reference control for descriptive comparison (Fig. 6). Cohort information, including sample numbers, disease/control status, and BioProject accession numbers, is provided in Table S2 and Fig. 6.

The relative abundance of the investigated *Faecalibacterium* species as seen in different diseases is shown in Fig. 6. At the genus level, *Faecalibacterium* showed a significant decrease across all three diseases, consistent with previous reports, but no significant differences were observed between disease groups. Among the dominant species, *F. taiwanense*, *F. prausnitzii*, and *F. duncaniae* exhibited similar reduction patterns compared with healthy controls. *F. hattorii* also showed a comparable decrease. By contrast, *F. longum*, despite its relatively high abundance within the genus, remained relatively stable across the different diabetes groups. This suggests that *F. longum* is relatively weakly associated with diabetes compared to *F. taiwanense*, *F. prausnitzii*, *F. duncaniae*, or *F. hattorii*.

Notably, several other *Faecalibacterium* species showed significant differences across diseases. *F. langellae* emerged as the most sensitive, with marked differences not only between health and disease, but also among disease groups, making it a promising indicator for disease characterization. *Faecalibacterium harmsenii* ranked second in sensitivity, followed by *F. butyricigenerans*, both showing significant abundance shifts across diseases, except between the two diabetes groups (Fig. 6). These disease-associated changes may reflect the sensitivity of the fastidious anaerobic genus *Faecalibacterium* to altered gut redox conditions and nutrient availability. Altogether, these findings highlight species-specific responses to host conditions and underscore the need for careful selection of *Faecalibacterium* species when using this genus as a biomarker of gut health.

## Conclusion

Here, we present the novel species *F. harmsenii* and the reclassification of another *Faecalibacterium* isolate as *F. langellae*. Genomic and phenotypic comparisons revealed distinct functional signatures in *F. harmsenii*, characterized by reduced stress tolerance, an expanded carbohydrate gene repertoire, and fewer defense systems, ARGs, and MGEs. Extending to the species level, we demonstrate species-specific ecological niches across hosts and differential sensitivities to human diseases, underscoring the need for species-level resolution in microbiome studies and highlighting the potential of *Faecalibacterium* species as refined biomarkers of gut health.

### Description of *F. harmsenii* sp. nov.

*Faecalibacterium harmsenii* (harm.sen’i.i. N.L. gen. Masc. n. *harmsenii*, of Harmsen) is named after the Dutch microbiologist Dr. Hermie J.M. Harmsen, in recognition of his contributions to the field of microbial physiology and gut microbiota research.

*Faecalibacterium harmsenii* cells are obligate anaerobic, Gram-stain-positive, and have a straight rod shape. The bacterium grows at 35–42°C (optimum, 37°C) and at pH 5.5–8.5 (optimum, pH 6.5–7.5). Growth is observed in broth containing 0–1% (w/v) NaCl. The type strain grows on cellobiose (weak), glucose, fucose, fructose, galactose, mannitol, mannose, lactose, sucrose, trehalose, rhamnose and inositol, but not on arabinose, maltose, raffinose, ribose and xylose. Positive enzyme reactions were obtained for acid phosphate, naphthol-AS-BI-phosphohydrolase, β- galactosidase, α-glucosidase and β-glucosidase, using the API ZYM system. The major fatty acids in cells cultured in YCFAG broth are C16:0, C15: 0, C16:0 DMA (dimethyl acetal), and C17:1ω6c.

The *F. harmsenii* type strain, HTF-05B^T^ (= NCIMB 15616^T^ =  DSM 35796^T^) was isolated from the feces of a healthy human donor. The DNA G + C content of the type strain is 58.5%. The GenBank accession numbers for the 16S rRNA gene sequence and the whole-genome sequence of this strain are PV864972 and GCF_051386655.1, respectively.

## Supplementary Material

Supplementary_material_ycag221

## Data Availability

All data generated during this study are included in this article and its supplementary information files. The accession numbers for strains HTF-05B^T^ and HTF-238 are as follows: GenBank accession numbers PV864972 and PV864973 for the 16S rRNA gene sequences, respectively, and genome assembly accession numbers GCF_051386655.1 and GCA_032584235.1 for the draft genomes, respectively. The type strain of *F. harmsenii*, HTF-05B^T^, has been deposited in DSMZ and NCIMB as DSM 35796^T^ and NCIMB 15616^T^, respectively, and is publicly available.
