# Supplementary material for: Faecalibacterium harmsenii sp. nov., an abundant but previously overlooked Faecalibacterium in the human gut
Source: ISME Commun. 2026 Jul 31;6(1):ycag221. doi: 10.1093/ismeco/ycag221 (PMC13540741; doi:10.1093/ismeco/ycag221)
Supplement: Supplementary file 1 [file supplementary_material_ycag221.zip › Supplementary_Materials_FigureS1.pdf]

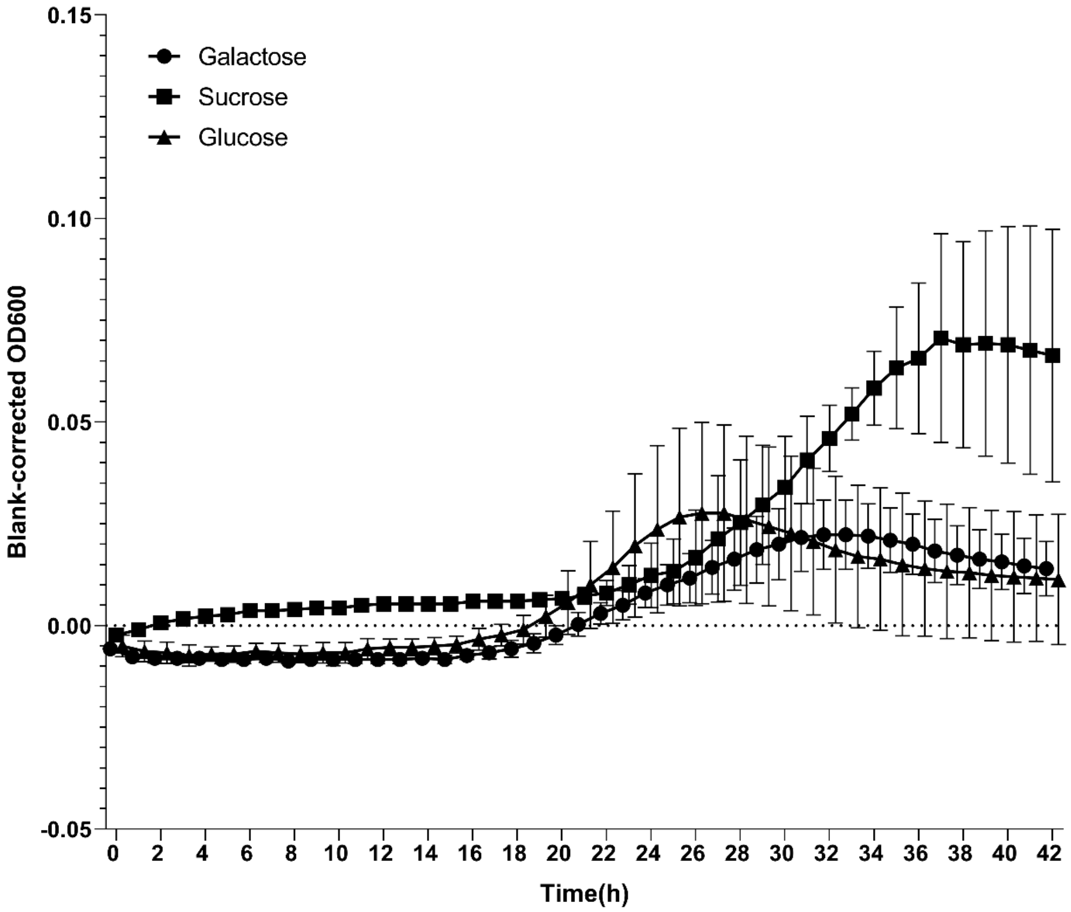

Figure S1. Growth of *F. harmsenii* strain HTF-05B cultured with different sugar substrates. Media blank controls were subtracted from OD_{600} values for each time point and condition. Therefore, the OD_{600} values in the y-axis are referred to as 'blank-corrected'. As a consequence of this correction, negative values occurred when the culture OD_{600} was slightly lower than the matched blank, likely reflecting low-OD background variation rather than negative growth.
