# Supplementary material for: Faecalibacterium harmsenii sp. nov., an abundant but previously overlooked Faecalibacterium in the human gut
Source: ISME Commun. 2026 Jul 31;6(1):ycag221. doi: 10.1093/ismeco/ycag221 (PMC13540741; doi:10.1093/ismeco/ycag221)
Supplement: Supplementary file 1 [file supplementary_material_ycag221.zip › Supplementary_Materials_FigureS2.pdf]

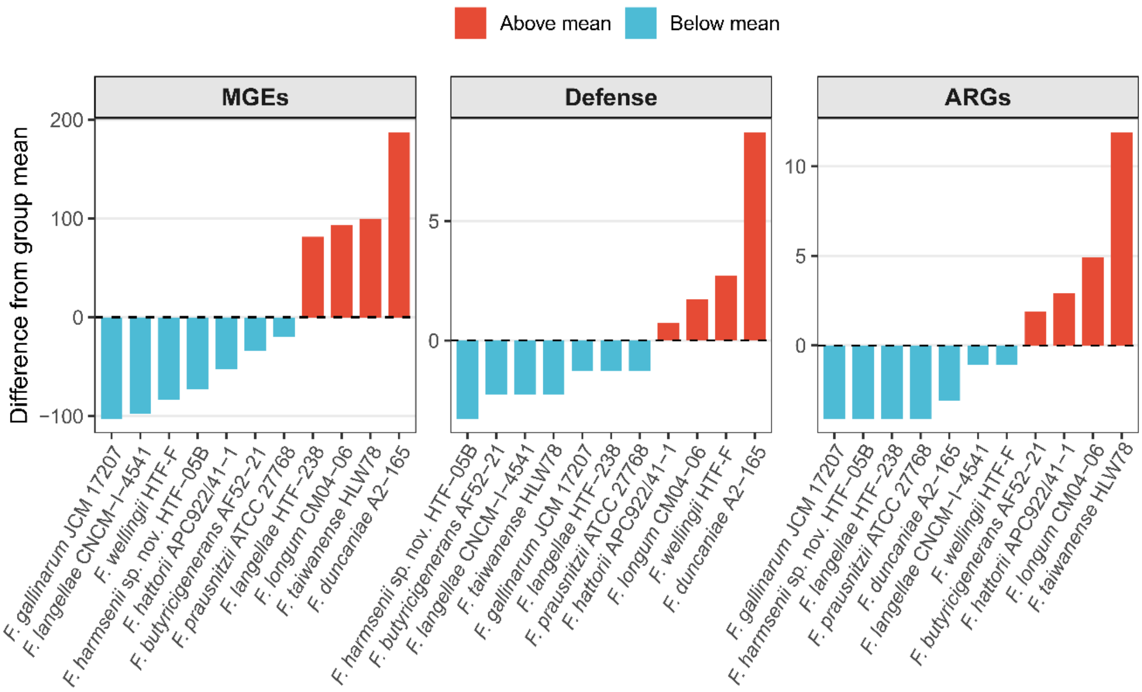

Figure S2. Comparison of MGEs, defense systems, and ARGs relative to the type-strain mean. Bar plots show the differences between each strain and the group mean for mobile genetic elements (MGEs), defense systems, and antibiotic resistance genes (ARGs). The mean value was calculated separately for each functional category across the type-strain panel. Bars above zero indicate values higher than the group mean, whereas bars below zero indicate values lower than the group mean. *F. harmsenii sp.* nov. HTF-05B showed below-average numbers of MGEs, defense systems, and ARGs compared with the overall type-strain panel.
