# Supplementary material for: Faecalibacterium harmsenii sp. nov., an abundant but previously overlooked Faecalibacterium in the human gut
Source: ISME Commun. 2026 Jul 31;6(1):ycag221. doi: 10.1093/ismeco/ycag221 (PMC13540741; doi:10.1093/ismeco/ycag221)
Supplement: Supplementary file 1 [file supplementary_material_ycag221.zip › Supplementary_Materials_FigureS3.pdf]

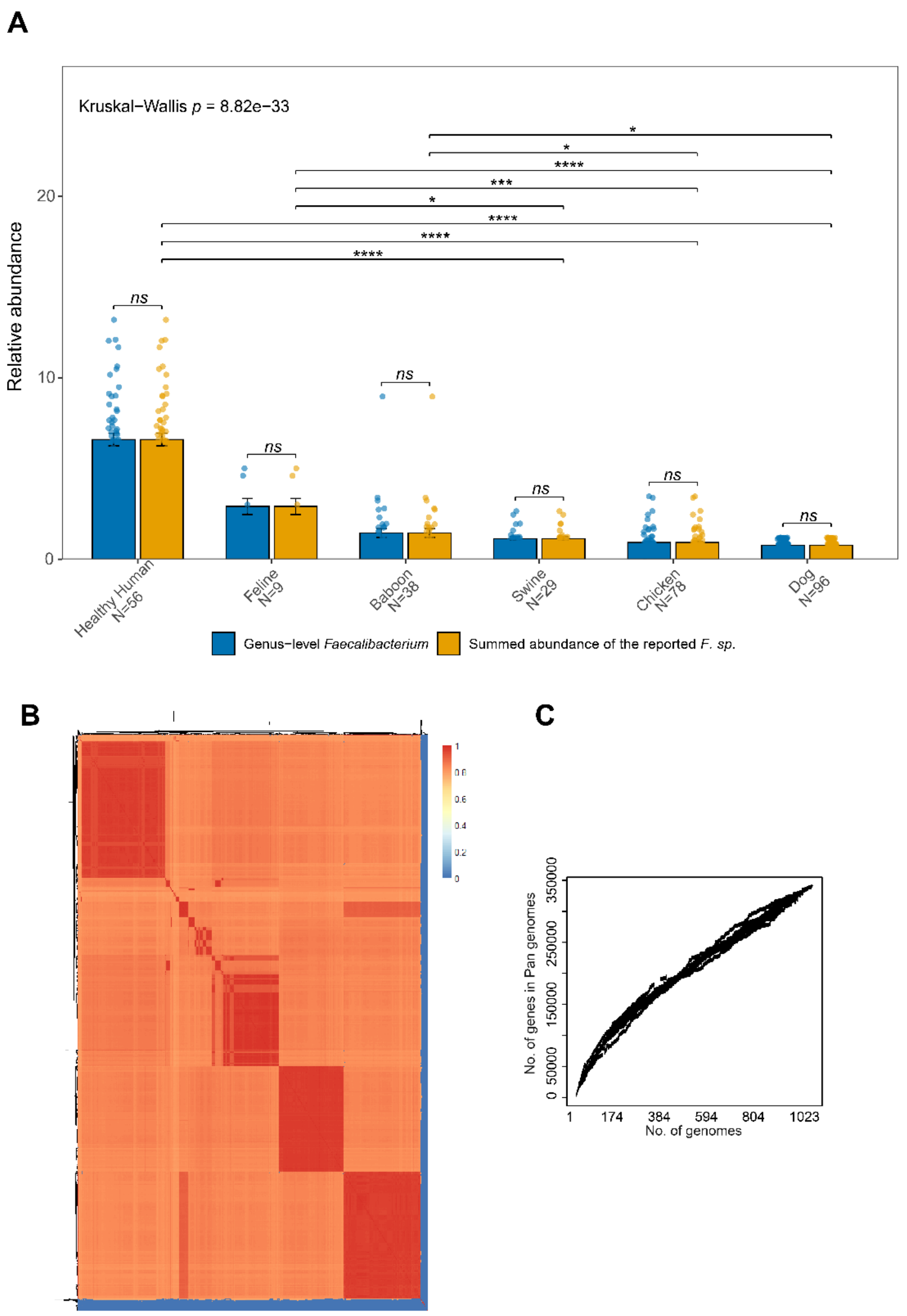

Figure S3. Ecological and genomic features of *Faecalibacterium* species. A. Comparison of *Faecalibacterium* genus-level abundance with the summed abundance of *Faecalibacterium* species across different host backgrounds. B. ANI matrix of all publicly available *Faecalibacterium* genomes in the NCBI Genome database (N = 1210). C. Pan-genome curves of *Faecalibacterium* constructed using Roary v3.13.0 with a 95% BLASTP identity threshold for orthologous gene clustering. It represents the cumulative number of non-redundant gene clusters.
