# Supplementary material for: Faecalibacterium harmsenii sp. nov., an abundant but previously overlooked Faecalibacterium in the human gut
Source: ISME Commun. 2026 Jul 31;6(1):ycag221. doi: 10.1093/ismeco/ycag221 (PMC13540741; doi:10.1093/ismeco/ycag221)
Supplement: Supplementary file 1 [file supplementary_material_ycag221.zip › Supplementary_Materials_TableS1.pdf]

Table S1. Genomic features of strains used in this study

Species	Strain	ID/assembly	Genome size (Mb)	G+C content (mol%)	N50	Numbers of Contigs (≥500 bp)	Completeness*	Contamination*	Genome coverage**
F. harmsenii sp. nov.	HTF-05B	GCF_051386655.1	2.8	58.5	190306	26	89.57%	3.51%	70.6452x
F. taiwanense	HLW78	GCF_036632915.2	3.0	55.9	2955389	1	91.32%	3.64%	496x
F. prausnitzii	ATCC 27768	GCF_003324185.1	3.0	56.4	136767	76	95.78%	3.8%	300x
F. duncaniae	A2-165	GCF_002734145.1	3.1	56.3	3110044	1	87.17%	6.74%	395.5x
F. hattorii	APC922/41-1	GCF_003287455.1	2.8	57.7	126007	54	87.36%	4.56%	726x
F. longum	CM04-06	CNA0017731	2.9	57.8	119299	47	99.32%	0%	-
F. wellingii	HTF-F	GCF_023347535.1	2.8	56.5	2776287	1	89.74%	4.46%	45x
F. butyricigenerans	AF52-21	CNA0017730	3.0	57.5	191233	73	100.00%	0%	-

F. gallinarum	JCM 17207	GCF_022180365.1	2.8	59.0	73159	76	80.66%	8.91%	2200x
F. langellae	CNCM I-4541	GCF_002549775.1	2.8	58.1	181717	78	87.45%	4.71%	1403x
F. langellae	HTF-238	GCA_032584235.1	3.1	57.5	245449	42	89.02%	4.3%	350x

*Note that *Faecalibacterium* genomes with >85% completeness and <5% contamination were retained for further analyses, with the exception of the designated type strains, which were included regardless of these thresholds. **Genome sequencing coverage has been included where available.
