# Supplementary material for: Faecalibacterium harmsenii sp. nov., an abundant but previously overlooked Faecalibacterium in the human gut
Source: ISME Commun. 2026 Jul 31;6(1):ycag221. doi: 10.1093/ismeco/ycag221 (PMC13540741; doi:10.1093/ismeco/ycag221)
Supplement: Supplementary file 1 [file supplementary_material_ycag221.zip › Supplementary_Materials_TableS2.pdf]

Table S2. Cohort and host database information used in this study

Cohort	BioProject number	Host-derived database
Chicken	PRJEB21076	GCF_016699485.2_bGalGal1.mat.broiler.GRCg7b_genomic.fna
Dog	PRJEB20308	GCF_000002285.5_Dog10K_Boxer_Tasha_genomic.fna
Cat	PRJNA758898	GCF_018350175.1_F.catus_Fca126_mat1.0_genomic.fna
Swine	PRJEB11755	GCF_000003025.6_Sscrofa11.1_genomic.fna
Cow	PRJEB21624	GCF_002263795.3_ARS-UCD2.0_genomic.fna
Baboon	PRJNA271618	GCF_008728515.1_Panubis1.0_genomic.fna
IBD (including healthy controls)	PRJNA400072	GRCh38_noalt_as
Type 2 diabetes	PRJEB15179	GRCh38_noalt_as
Gestational diabetes mellitus	PRJNA853814	GRCh38_noalt_as
