# Supplementary material for: Faecalibacterium harmsenii sp. nov., an abundant but previously overlooked Faecalibacterium in the human gut
Source: ISME Commun. 2026 Jul 31;6(1):ycag221. doi: 10.1093/ismeco/ycag221 (PMC13540741; doi:10.1093/ismeco/ycag221)
Supplement: Supplementary file 1 [file supplementary_material_ycag221.zip › Supplementary_Materials_TableS3.pdf]

Table S3. dDDH (top, bold type) and ANIb (bottom, normal type) values (%) within the genus *Faecalibacterium*

			1	2	3	4	5	6	7	8	9	10	11
1	*F. harmsenii sp. nov.*	HTF-05B	-	**30.9**	**30.9**	**28.9**	**28**	**28.1**	**30.0**	**28.2**	**22.9**	**31.0**	**32.2**
2	*F. taiwanense*	HLW78	85.6	-	**30.9**	**29.3**	**28.5**	**28.3**	**45.5**	**29.0**	**22.8**	**34.1**	**34.9**
3	*F. prausnitzii*	ATCC 27768	85.7	85.6	-	**28.6**	**28.5**	**29.4**	**30.5**	**28.4**	**22.8**	**29.9**	**31.1**
4	*F. duncaniae*	A2-165	84.2	83.9	83.9	-	**29.8**	**28.3**	**28.3**	**28.8**	**23.1**	**28.1**	**28.8**
5	*F. hattorii*	APC922/41-1	83.7	83.7	84.1	85.1	-	**29.4**	**27.3**	**28.4**	**22.6**	**27.6**	**28.4**
6	*F. longum*	CM04-06	83.7	83.1	83.5	83.9	84.1	-	**27.2**	**41.8**	**23.3**	**27.8**	**28.2**
7	*F. wellingii*	HTF-F	85.5	91.4	85.5	83.8	83.4	82.9	-	**28.0**	**23.3**	**33.8**	**34.3**
8	*F. butyricigenerans*	AF52-21	83.5	83.7	84.0	83.6	84.7	90.4	83.1	-	**23.9**	**27.5**	**28.5**
9	*F. gallinarum*	JCM 17207	79.1	78.5	78.8	79.0	79.0	79.4	78.5	79.4	-	**22.7**	**22.6**
10	*F. langellae*	CNCM I-4541	86.0	87.5	85.4	83.6	83.7	83.5	87.1	83.5	78.9	-	**77.9**
11	*F. langellae*	HTF-238	86.8	87.8	86.4	84.5	84.5	83.9	87.2	84.3	78.7	97.3	-

Note: Species boundaries were interpreted using ANI ≥95–96% and dDDH ≥70% as commonly accepted thresholds. HTF-238 exceeded these thresholds with *F. langellae* CNCM I-4541, whereas HTF-05B remained below these thresholds against all described *Faecalibacterium* type strains, supporting its designation as *F. harmsenii sp*. nov. The genomes listed in this Table were retrieved from the NCBI Genome database according to their accession numbers, as shown in Table S1.
