# Supplementary material for: Faecalibacterium harmsenii sp. nov., an abundant but previously overlooked Faecalibacterium in the human gut
Source: ISME Commun. 2026 Jul 31;6(1):ycag221. doi: 10.1093/ismeco/ycag221 (PMC13540741; doi:10.1093/ismeco/ycag221)
Supplement: Supplementary file 1 [file supplementary_material_ycag221.zip › Supplementary_Materials_TableS4.pdf]

Table S4. Essential growth requirements and individual carbon source utilization profiles of *Faecalibacterium* strains

	F. harmsenii sp. nov. HTF-05B	*F. langellae* HTF-238	*F. prausnitzii* ATCC27768	*F. duncaniae* A2-165
L-arabinose	-	-	-	-
Cellobiose	w	+	w	+
Glucose	+	+	+	+
Fucose	+	-	w	-
Fructose	+	+	+	+
Galactose	+	+	+	+
Maltose	-	+	+	+
Mannitol	+	-	w	-
Mannose	+	W	w	-
Lactose	+	+	+	+
Raffinose	-	-	-	w
Ribose	-	-	-	-
Sucrose	+	W	+	+
Xylose	-	-	-	-
Trehalose	+	-	w	-
L-Rhamnose	+	W	+	-
Inositol	+	-	w	-
pH	5.5-8.5	6.0-8.0	5.5-7.5	5.5-7.5
Salinity	0–1 % (w/v) NaCl	0–1 % (w/v) NaCl	0–1 % (w/v) NaCl	0–1 % (w/v) NaCl
Temperature	35–42 °C	30–42°C	30–42°C	30–42°C

+, positive utilization; -, negative utilization; w, weak utilization.
