# Supplementary material for: Faecalibacterium harmsenii sp. nov., an abundant but previously overlooked Faecalibacterium in the human gut
Source: ISME Commun. 2026 Jul 31;6(1):ycag221. doi: 10.1093/ismeco/ycag221 (PMC13540741; doi:10.1093/ismeco/ycag221)
Supplement: Supplementary file 1 [file supplementary_material_ycag221.zip › Supplementary_Materials_TableS5.pdf]

Table S5. Differential characteristics among the tested *Faecalibacterium* strains.

Characteristic	*F. harmsenii* HTF-05B	*F. langellae* HTF-238	*F. langellae* CNCM I-4541	*F. prausnitzii* ATCC 27768
Enzyme activity:				
beta- glucosidase	+	-	+	+
alpha- glucosidase	+	+	-	+
beta- glucuronidase	-	w	-	+
Esterase (C4)	-	-	-	+
Major cellular fatty acids	C16:0, C15: 0, C16:0 DMA, C17:1ω6c	C16:0, C15: 0, C16:0 DMA, C17:1ω6c, C18:1ω7c DMA	C16:0, C16:0 DMA, C14:0, C18:1ω7c DMA	C14: 0, C15: 0, C16: 0

Note: +, positive reaction; −, negative reaction; w, weak reaction.
