# Supplementary material for: Faecalibacterium harmsenii sp. nov., an abundant but previously overlooked Faecalibacterium in the human gut
Source: ISME Commun. 2026 Jul 31;6(1):ycag221. doi: 10.1093/ismeco/ycag221 (PMC13540741; doi:10.1093/ismeco/ycag221)
Supplement: Supplementary file 1 [file supplementary_material_ycag221.zip › Supplementary_Materials_TableS6.pdf]

Table S6. Relative abundance of the top 20 KEGG functional categories across *Faecalibacterium* strains

KEGG pathway	*F. prausnitzii* ATCC 27768	*F. butyricigenerans* AF52-21	*F. duncaniae* A2-165	*F. gallinarum* JCM 17207	*F. hattorii* APC922/41-1	*F. langellae* CNCM I-4541	*F. longum* CM04-06	*F. taiwanense* HLW78	*F. wellingii* HTF-F	*F. harmsenii* HTF-05B	*F. langellae* HTF-238
Carbohydrate metabolism	9.24	8.89	8.43	10.87	8.60	8.88	8.65	9.40	8.77	10.43	8.85
Energy metabolism	3.97	3.89	3.88	3.71	3.89	4.06	3.94	3.94	3.87	3.99	3.93
Lipid metabolism	2.21	1.90	2.41	2.31	1.99	2.16	1.99	2.24	2.07	2.15	2.07
Nucleotide metabolism	2.79	2.92	2.45	2.62	2.85	2.57	2.90	2.91	2.79	2.96	2.46
Amino acid metabolism	6.31	6.46	6.06	6.55	6.20	6.76	5.84	6.27	6.66	6.14	6.34
Glycan biosynthesis and metabolism	3.83	3.89	3.48	4.19	3.26	3.07	3.24	3.00	3.37	3.49	3.15
Metabolism of cofactors and vitamins	4.37	3.89	3.97	3.45	4.07	4.19	3.85	4.25	4.32	4.39	4.14
Biosynthesis of other secondary metabolites	1.67	1.68	1.47	2.10	1.67	1.49	1.38	1.39	1.48	1.84	1.51
Translation	3.52	3.45	3.43	3.40	3.53	3.56	3.37	3.49	3.51	3.49	3.37

KEGG pathway	*F. prausnitzii* ATCC 27768	*F. butyricigenerans* AF52-21	*F. duncaniae* A2-165	*F. gallinarum* JCM 17207	*F. hattorii* APC922/41-1	*F. langellae* CNCM I-4541	*F. longum* CM04-06	*F. taiwanense* HLW78	*F. wellingii* HTF-F	*F. harmsenii* HTF-05B	*F. langellae* HTF-238
Folding, sorting and degradation	1.76	1.64	1.74	1.48	1.76	1.76	1.73	1.79	1.75	1.79	1.77
Replication and repair	2.75	2.65	3.03	2.92	2.85	2.71	2.72	2.69	2.83	2.78	2.68
Membrane transport	4.73	5.09	5.75	5.41	5.07	5.59	6.01	4.97	4.50	5.37	5.91
Signal transduction	2.12	2.57	1.65	1.79	2.13	2.03	2.42	2.46	2.61	1.70	2.11
Cellular community - prokaryotes	2.12	2.61	2.32	1.96	2.49	2.57	2.68	2.55	2.29	2.24	2.55
Protein families: metabolism	6.85	6.68	7.22	6.90	7.19	6.81	6.62	6.80	6.79	6.85	6.65
Protein families: genetic information processing	20.50	19.91	19.66	18.77	20.23	20.15	19.42	19.92	20.11	19.53	19.85
Protein families: signaling and cellular processes	15.05	16.06	16.94	15.84	16.38	15.55	17.13	15.98	16.10	15.00	16.44

KEGG pathway	*F. prausnitzii* ATCC 27768	*F. butyricigenerans* AF52-21	*F. duncaniae* A2-165	*F. gallinarum* JCM 17207	*F. hattorii* APC922/41-1	*F. langellae* CNCM I-4541	*F. longum* CM04-06	*F. taiwanense* HLW78	*F. wellingii* HTF-F	*F. harmsenii* HTF-05B	*F. langellae* HTF-238
Unclassified: metabolism	3.02	2.61	2.54	2.92	2.58	2.84	2.72	2.82	2.79	2.69	2.81
Unclassified: signaling and cellular processes	1.35	1.42	1.69	1.35	1.49	1.49	1.43	1.39	1.53	1.48	1.55
Poorly characterized	1.85	1.77	1.87	1.44	1.76	1.76	1.95	1.75	1.84	1.70	1.86

Note: Values indicate the relative abundance (%) of genes assigned to each KEGG functional category among all KEGG-annotated genes in each genome. This Table provides a descriptive comparison across representative genomes. Therefore, no inferential statistical test was applied.
