# Supplementary material for: Faecalibacterium harmsenii sp. nov., an abundant but previously overlooked Faecalibacterium in the human gut
Source: ISME Commun. 2026 Jul 31;6(1):ycag221. doi: 10.1093/ismeco/ycag221 (PMC13540741; doi:10.1093/ismeco/ycag221)
Supplement: Supplementary file 1 [file supplementary_material_ycag221.zip › Supplementary_Materials_TableS7.pdf]

Table S7. The ARGs in *Faecaliabcterium* type strains

# Antimicrobial	Genetic background	Location	Strain
lincomycin	erm(B) (erm(B)_X72021)	Chromosome	*F. butyricigenerans* AF52-2
clindamycin	erm(B) (erm(B)_X72021)	Chromosome	*F. butyricigenerans* AF52-2
erythromycin	erm(B) (erm(B)_X72021)	Chromosome	*F. butyricigenerans* AF52-2
guinupristin	erm(B) (erm(B)_X72021)	Chromosome	*F. butyricigenerans* AF52-2
pristinamycin ia	erm(B) (erm(B)_X72021)	Chromosome	*F. butyricigenerans* AF52-2
virginiamycin	erm(B) (erm(B)_X72021)	Chromosome	*F. butyricigenerans* AF52-2
Streptomycin	ant(6)-Ia (ant(6)-Ia_KF864551)	Chromosome	*F. duncaniae* A2165
Amoxicillin	blaTEM-116 (blaTEM-116_AY425988)	Chromosome	*F. hattorii* APC922
Ampicillin	blaTEM-116 (blaTEM-116_AY425988)	Chromosome	*F. hattorii* APC922
piperacillin	blaTEM-116 (blaTEM-116_AY425988)	Chromosome	*F. hattorii* APC922
ticarcillin	blaTEM-116 (blaTEM-116_AY425988)	Chromosome	*F. hattorii* APC922
cephalothin	blaTEM-116 (blaTEM-116_AY425988)	Chromosome	*F. hattorii* APC922
tetracycline	tet(C) (tet(C)_AF055345)	Chromosome	*F. hattorii* APC922
doxycycline	tet(C) (tet(C)_AF055345)	Chromosome	*F. hattorii* APC922
tetracycline	tet(W) (tet(W)_AJ427422)	Chromosome	*F. langellae* CNCM-I-4541
doxycycline	tet(W) (tet(W)_AJ427422)	Chromosome	*F. langellae* CNCM-I-4541
minocycline	tet(W) (tet(W)_AJ427422)	Chromosome	*F. langellae* CNCM-I-4541
gentamicin	aac(6')-aph(2'') (aac(6')-aph(2'')_M13771)	Chromosome	*F. longum* CM04-06
tobramycin	aac(6')-aph(2'') (aac(6')-aph(2'')_M13771)	Chromosome	*F. longum* CM04-06

# Antimicrobial	Genetic background	Location	Strain
streptomycin	aac(6')-aph(2'') (aac(6')-aph(2'')_M13771), ant(6)-Ia (ant(6)-Ia_KF864551)	Chromosome	*F. longum* CM04-06
amikacin	aac(6')-aph (2'') (aac(6')-aph(2'')_M13771)	Chromosome	*F. longum* CM04-06
isepamicin	aac(6')-aph(2'') (aac(6')-aph(2'')_M13771)	Chromosome	*F. longum* CM04-06
dibekacin	aac(6')-aph(2'') (aac(6')-aph(2'')_M13771)	Chromosome	*F. longum* CM04-06
kanamycin	aac(6')-aph(2'') (aac(6')-aph(2'')_M13771)	Chromosome	*F. longum* CM04-06
netilmicin	aac(6')-aph(2'') (aac(6')-aph(2'')_M13771)	Chromosome	*F. longum* CM04-06
fortimicin	aac(6')-aph(2'') (aac(6')-aph(2'')_M13771)	Chromosome	*F. longum* CM04-06
lincomycin	erm(B) (erm(B)_U18931), cfr(C) (cfr(C)_CANB01000378)	Chromosome	*F. taiwanense* HLW78
clindamycin	erm(B) (erm(B)_U18931), cfr(C) (cfr(C)_CANB01000378)	Chromosome	*F. taiwanense* HLW78
dalfopristin	cfr(C) (cfr(C)_CANB01000378)	Chromosome	*F. taiwanense* HLW78
pristinamycin iia	cfr(C) (cfr(C)_CANB01000378)	Chromosome	*F. taiwanense* HLW78
virginiamycin m	cfr(C) (cfr(C)_CANB01000378)	Chromosome	*F. taiwanense* HLW78
tiamulin	cfr(C) (cfr(C)_CANB01000378)	Chromosome	*F. taiwanense* HLW78
erythromycin	erm(B) (erm(B)_U18931)	Chromosome	*F. taiwanense* HLW78
tetracycline	tet(W) (tet(W)_FN396364)	Chromosome	*F. taiwanense* HLW78
doxycycline	tet(W) (tet(W)_FN396364)	Chromosome	*F. taiwanense* HLW78
minocycline	tet(W) (tet(W)_FN396364)	Chromosome	*F. taiwanense* HLW78
quinupristin	erm(B) (erm(B)_U18931)	Chromosome	*F. taiwanense* HLW78

# Antimicrobial	Genetic background	Location	Strain
pristinamycin ia	erm(B) (erm(B)_U18931)	Chromosome	*F. taiwanense* HLW78
virginiamycin s	erm(B) (erm(B)_U18931)	Chromosome	*F. taiwanense* HLW78
linezolid	cfr(C) (cfr(C)_CANB01000378)	Chromosome	*F. taiwanense* HLW78
chloramphenicol	cfr(C) (cfr(C)_CANB01000378)	Chromosome	*F. taiwanense* HLW78
florfenicol	cfr(C) (cfr(C)_CANB01000378)	Chromosome	*F. taiwanense* HLW78
tetracycline	tet(W) (tet(W)_AJ427422)	Chromosome	*F. wellingii* HTF-F
doxycycline	tet(W) (tet(W)_AJ427422)	Chromosome	*F. wellingii* HTF-F
minocycline	tet(W) (tet(W)_AJ427422)	Chromosome	*F. wellingii* HTF-F
